# Epsilon PKC Increases Brain Mitochondrial SIRT1 Protein Levels via Heat Shock Protein 90 following Ischemic Preconditioning in Rats

**DOI:** 10.1371/journal.pone.0075753

**Published:** 2013-09-13

**Authors:** John W. Thompson, Kunjan R. Dave, Isabel Saul, Srinivasan V. Narayanan, Miguel A. Perez-Pinzon

**Affiliations:** Cerebral Vascular Disease Research Laboratories, Department of Neurology, Miller School of Medicine, University of Miami, Miami, Florida, United States of America; National University of Singapore, Singapore

## Abstract

Ischemic preconditioning is a neuroprotective mechanism whereby a sublethal ischemic exposure is protective against a subsequent lethal ischemic attack. We previously demonstrated that SIRT1, a nuclear localized stress-activated deacetylase, is vital for ischemic preconditioning neuroprotection. However, a recent study demonstrated that SIRT1 can also localize to the mitochondria. Mitochondrial localized SIRT1 may allow for a direct protection of mitochondria following ischemic preconditioning. The objective of this study was to determine whether ischemic preconditioning increases brain mitochondrial SIRT1 protein levels and to determine the role of PKCɛ and HSP90 in targeting SIRT1 to the mitochondria. Here we report that preconditioning rats, with 2 min of global cerebral ischemia, induces a delayed increase in non-synaptic mitochondrial SIRT1 protein levels which was not observed in synaptic mitochondria. This increase in mitochondrial SIRT1 protein was found to occur only in neuronal cells and was mediated by PKCε activation. Inhibition of HSP90, a protein chaperone involved in mitochondrial protein import, prevented preconditioning induced increases in mitochondrial SIRT1 and PKCε protein. Our work provides new insights into a possible direct role of SIRT1 in modulating mitochondrial function under both normal and stress conditions, and to a possible role of mitochondrial SIRT1 in activating preconditioning induced ischemic tolerance.

## Introduction

Ischemic preconditioning (IPC) is an innate neuroprotective mechanism in which a mild ischemic stress protects against a subsequent lethal ischemic exposure. IPC activates both early (0-3h) and delayed (24-72 h) windows of protection [1-3]. The early window of protection is mediated by a rapid post-translational modification of proteins, whereas, the delayed window of protection is mediated by alterations in gene expression [4-6]. PKC epsilon (PKCɛ) is a novel member of the protein kinase C family which has been demonstrated by our laboratory and others to be pivotal in IPC mediated neuroprotection [7,8]. Our laboratory has demonstrated that activation of PKCɛ, in the absence of IPC, is sufficient to activate neuroprotection, whereas, inhibition of PKCε blunts IPC mediated neuroprotection against cerebral ischemia [8-10].

Sirtuin 1 (SIRT1) is a member of the sirtuin family of NAD^+^ dependent deacetylases which is implicated as a metabolic sensor of the cell [11-13]. SIRT1 is neuroprotective in numerous models of neurodegenerative diseases including ischemia/reperfusion [14-16]. In brain derived endothelial cells, SIRT1 protects against oxygen and glucose deprivation induced cell death [17]. SIRT1 is primarily localized to the nucleus where it regulates gene transcription through deacetylation of histone and non-histone proteins [18]. It has been demonstrated in both the brain and heart that IPC activates SIRT1 leading to ischemic tolerance. We have previously demonstrated that IPC activates nuclear SIRT1 deacetylase activity and SIRT1 mediated neuroprotection against cerebral ischemia [19,20].

Although SIRT1s function has been primarily characterized in the nucleus, a recent study has demonstrated that SIRT1 can also localize to the mitochondria. Aquilano et al. [21] demonstrated that in the mouse brain, liver and muscle SIRT1 was localized to the mitochondrial matrix where it interacted with mitochondrial DNA and the transcription factor TFAM. The association of SIRT1 with mitochondrial DNA and transcription factors suggests a transcriptional regulatory role of SIRT1 in the mitochondria; similar to its described nuclear activities. However, it is currently not known if mitochondrial SIRT1 protein levels are altered during periods of stress which might have a direct effect on mitochondrial function. Since SIRT1 can localize to mitochondria, and IPC is known to protect brain mitochondria, we hypothesized that IPC increases mitochondrial SIRT1 protein levels. Therefore the objective of this study was to determine the effects of IPC on mitochondrial SIRT1 protein levels and the role of PKCε and heat shock protein 90 (HSP90) in targeting SIRT1 to the mitochondria.

## Materials and Methods

### Ethics statement

All animal protocols were approved by the Animal Care and Use Committee of the University of Miami (assurance number: A-3224-01). All experiments were conducted in accordance to ARRIVE guidelines. Male (250-300g) and 16-17 day-pregnant Sprague-Dawley rats were purchased from Charles Rivers Laboratories and housed in a temperature controlled environment with 12 hr light -12 h dark cycle and *ad libitum* food and water.

### Materials

Minimum Essential Medium (MEM), Hanks Balanced Salt Solution (HBSS) and Fetal Bovine Serum (FBS) were purchased from Gibco/Life Technologies (Grand Island, NY). The HSP90 inhibitor 17-AAG was purchased from TOCRIS Bioscience (Bristol, UK). The PKCε activator, ψεRACK, and its inhibitor, εV1-2 peptides, and TAT control peptide were purchased from KAI Pharmaceuticals Inc (San Francisco, CA). All other reagents were purchased from Sigma (St. Louis, MO) unless otherwise noted.

### Animal model of global cerebral ischemia

Global cerebral ischemia was induced in male Sprague Dawley rats by bilateral carotid occlusion with systemic hypotension as previously described [22]. In brief, rats were anesthetized with isoflurane and 70% nitrous oxide (with a balance of oxygen) and both common carotid arteries exposed and dissected free from the surrounding tissue. A ligature consisting of polyethylene (PE-10) tubing encased in SILASTIC tubing was placed around each artery. Sublethal ischemic stress (IPC) was induced by gradually withdrawing blood from the femoral vein into a heparinized syringe until systemic blood pressure reached 50 mmHg, followed by tightening of the carotid ligatures bilaterally for 2 min. Cerebral ischemia was terminated by removal of the carotid ligatures and replacement of the shed blood into the femoral vein thereby restoring the mean arterial blood pressure to pre-ischemic levels. Brain and body temperature were maintained at 37°C throughout the experiment. Sham animals underwent the same treatment as described above but without IPC induction. Animals were treated with buprenorphine for pain management. Hippocampal synaptic and non-synaptic mitochondria were isolated after 2 or 48 hrs of reperfusion as previously described [23]. In brief, hippocampi were homogenized in 250 mM sucrose, 1 mM BSA, 0.25 mM DTT and 1 mM EDTA, pH 7.4 and the resulting homogenate centrifuged at 500 x g for 5 min to remove the nuclear fraction. The resulting supernatant was layered onto a Percoll gradient and the synaptosomes and non-synaptic mitochondria isolated by centrifugation at 32,500 x g for 5 min. The synaptosomal layer and non-synaptic mitochondria were removed from the Percoll gradient, washed with homogenization buffer and resuspended in 0.25 M sucrose. Synaptosomal mitochondria were isolated by nitrogen cell bomb (1,200 psi for 7.5 min) rupturing of the synaptosomes. Synaptic mitochondria were pelleted at 20,000 x g for 20 min at 4°C.

### Preparation of cortical neuronal and glial cell cultures

Primary cortical glial and neuronal only cultures were prepared as previously described [10]. In brief, neuronal only cultures were prepared from the cortices of E18-19 day pups. Embryos were harvested by caesarian section and the cortices rapidly removed and placed in ice-cold HBSS. The tissue was enzymatically digested for 15 min at 37°C. The resulting cellular suspension was filtered thru a 70 µm cell strainer and plated at a density of 3.5 hemispheres per plate in MEM supplemented with 5% FBS, 5 mM GlutaMax (InVitrogen/Life Technologies, Grand Island, NY) and 15 mM glucose. Glial only cultures were prepared from the cortices of 1-2 day old pups. Glial cells were isolated as described above for neuronal only cultures and plated in the same media. The cells were used for experimentation after 14 days in culture.

### Oxygen and glucose deprivation preconditioning

To mimic sublethal cerebral ischemia, cells were exposed to oxygen and glucose deprivation (OGD) for 45 min [10]. To induce OGD, cells were washed two times with glucose-free HBSS (CaCl_2_. 2H_2_O 1.26 mM, KCl 5.37 mM, KH_2_PO_4_ 0.44 mM, MgCl_2_ 0.49 mM, MgSO_4_·7H_2_O 0.41 mM, NaCl 136.9 mM, NaHCO_3_ 4.17 mM, Na_2_HPO_4_·7H_2_O 0.34 mM, sucrose 15 mM, pH 7.4) and exposed to an oxygen-free environment (90% nitrogen, 5% hydrogen, and 5% CO_2_, 37°C) using a COY anaerobic chamber (COY Laboratory Products Inc, Lake Charter Township, MI). OGD was terminated by placing the cells back into normal aerobic media.

### Subcellular fractionation

Subcellular fractionation was performed as previously described [24-26]. For mitochondrial isolation, cells were suspended in 10 mM Tris, pH 7.4, and 320 mM sucrose, 1 mM EDTA, 1 mM DTT with protease and phosphatase inhibitors and homogenized using a teflon-glass homogenizer. The resulting homogenate was centrifuged at 1,500 x g for 5 min at 4°C. The supernatant was further centrifuged at 10,000 x g for 20 min at 4°C to pellet mitochondria. Mitochondrial pellets were washed three times with isolation buffer and suspended in RIPA lysis buffer for Western blot analysis or in isolation buffer for mitoplast generation and stored at -80°C. Mitoplasts were prepared by incubating mitochondria in 5 vol of hypotonic buffer (10 mM Tris, pH 7.4, 1 mM EDTA, and 1 mM DTT) for 1 hr on ice followed by incubation with 150 mM KCl for an additional 2 min. The mitoplasts were separated from postmitoplast supernatants by centrifugation at 20,000 x g for 20 min at 4°C. For alkali extraction, mitochondria were incubated for 30 min on ice with freshly prepared 0.1 M sodium carbonate (pH ~ 11.5). The samples were centrifuged at 40,000 x g for 1 hr at 4°C and the resulting pellet resuspended in sample buffer. To isolate the cytoplasmic fraction, cells were suspended in 10 mM Tris, (pH 7.4), 320 mM sucrose, 1 mM EDTA, 1 mM DTT with protease and phosphatase inhibitors and homogenized using a teflon-glass homogenizer. The cytoplasmic fraction was isolated from the nuclear and mitochondrial fractions by centrifugation at 10,000 g for 20 min to pellet nuclei and mitochondria. To isolate the nuclear fraction, cells were resuspended in buffer consisting of 20 mM Tris (pH 8.0), 137 mM NaCl, 0.5 mM EDTA, 10% glycerol, and 1 mM DTT for 10 min on ice. Cells were lysed with 1% Nonidet P-40, and vortexed vigorously for 10 secs followed by centrifugation at 10,000 x g. for 10 min [10,27]. The resulting pellet consisting of enriched nuclei was lysed with RIPA buffer.

### Western blotting

Western blots were performed as previously described [10,27]. Cells and mitochondria were lysed in RIPA Buffer (20 mM Tris-HCl (pH 7.5), 150 mM NaCl, 1 mM EDTA, 1% NP-40, 1% sodium deoxycholate, 2.5 mM sodium pyrophosphate, 1 mM Na _3_VO_4_ and 1 mM PMSF). Protein concentration was determined by BCA protein assay and 40 µg of protein was loaded onto an 8 or 12% SDS-polyacrylamide gel and electroblotted to nitrocellulose. Membranes were blocked in 5% dry milk/TBST and hybridized with primary antibodies overnight at 4°C. Blots were probed with PKCε (Santa Cruz Biotechnology, Dallas, TX), SIRT1 (Santa Cruz Biotechnology), HSP60 (Cell Signaling Technology, Danvers, MA), acetylated lysine (Cell Signaling Technology), β-actin (Sigma), lamin-B (Cell Signaling Technology), cytochrome C (BD Pharmingen, San Jose, CA) and COXIV (InVitrogen/Life Technologies). Membranes were washed with TBST followed by incubation with secondary antibodies (Pierce, Thermo Scientific; Rockford, IL) for 1 hr at room temperature. Proteins were detected using enhanced chemiluminescence (ECL) system (Pierce, Thermo Scientific). Western blot densitometry was analyzed using ImageJ software from NIH.

### SIRT1 activity assay

Mitochondrial SIRT1 activity was determined using a kit from Cayman Chemicals (Ann Arbor, MI) as previously described [28]. In brief, mitochondria were lysed in IP lysis buffer consisting of 1% Nonidet P-40, 20 mM Tris (pH 8.0) 137 mM NaCl, 0.5 mM EDTA, 10% glycerol, 1 mM phenylmethylsulfonyl fluoride and protease and phosphatase inhibitors (Roche) and the lysate (500 µg) incubated with 2 µg of immunoprecipitating SIRT1 antibody (Santa Cruz Biotechnology) overnight at 4°C followed by precipitation with protein G Sepharose beads (Sigma) for 2 hrs at 4°C. The pellet was washed 4 x with IP lysis buffer followed by incubation with SIRT1 activity reaction mixture for 1 hr at room temperature with shaking. SIRT1 deacetylase activity was determined as per the manufacturer’s instructions.

### Mitochondrial respiration

Mitochondrial respiration studies were conducted as previously described [23]. In brief, cortical synaptic and non-synaptic mitochondria were isolated from either naïve rats or from rats treated with the PKCε activator peptide (ψεRACK; 0.2 mg/kg i.p.) [29,30] or the TAT carrier peptide. The isolated mitochondria were treated with the general sirtuin inhibitor, sirtinol (1µM), the specific SIRT1 inhibitor, EX527 (10 µM [31]), or DMSO for 30 min on ice. The rate of mitochondrial oxygen consumption was determined using a Clark-type oxygen electrode in the presence of 0.5 mM ADP and the following substrates: 1) pyruvate and malate (induces mitochondrial respiration at complex I); 2) succinate and glycerol-3-phosphate (induces mitochondrial respiration at complex II); and 3) ascorbate and *N,N,N*’,N’-tetramethyl-*p-*phenylenediamine (TMPD) (induces mitochondrial respiration at complex IV).

### Statistical analysis

All data are expressed as mean ± SEM. Statistical analysis between two groups was performed using the unpaired Student’s *t*-test. Statistical analysis between more than two groups was performed using a one-way ANOVA with Dunnett’s multiple comparison post hoc test. *P* < 0.05 was considered statistically significant.

## Results

### Ischemic preconditioning increases mitochondrial SIRT1 protein levels

**Figure 1:**
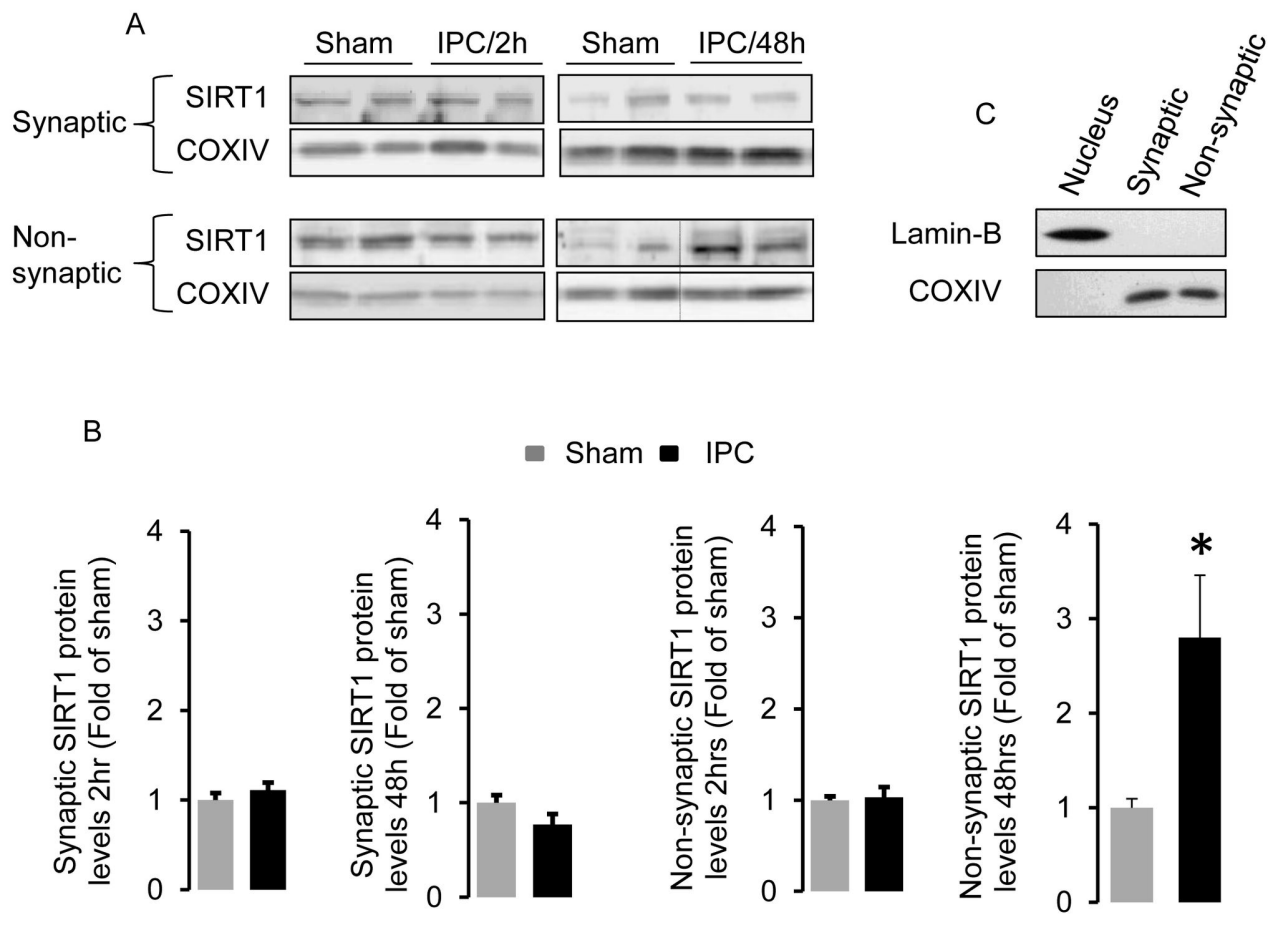
Mitochondrial SIRT1 protein levels increase in the hippocampus following IPC. In (A) a representative Western blot showing SIRT1 protein levels in hippocampal synaptic and non-synaptic mitochondria at 2 and 48 hrs following 2 min of global cerebral ischemia (IPC) from two different animals. Western blot quantitation of synaptic and non-synaptic mitochondrial SIRT1 protein levels is shown in (B) (n = 8). In (C) mitochondrial purity from nuclear contamination was determined by western blot analysis using antibodies to the nuclear localized protein Lamin-B and to the mitochondrial localized protein COXIV. Data are means ± SEM compared to sham treated animals. * *p* < 0.05 increase from sham by Student’s *t*-test.

To determine if IPC alters mitochondrial levels of SIRT1, we exposed rats to 2 min of global cerebral ischemia with systemic hypotension to induce IPC. SIRT1 protein levels were determined in hippocampal synaptic and non-synaptic mitochondria 2 and 48 hrs later. As demonstrated in Figure 1A and B, mitochondrial SIRT1 protein levels were unchanged 2 hrs following IPC exposure. However, at 48 hrs of reperfusion there was a significant increase (*p* < 0.05) in non-synaptic mitochondrial SIRT1 protein levels. In contrast, there was no change in synaptic mitochondrial SIRT1 levels at any of the time points examined. Mitochondrial purity was confirmed using the nuclear marker lamin-B (Figure 1C). The delayed increase in mitochondrial SIRT1 protein levels exhibited a temporal profile similar to that previously demonstrated in nuclear SIRT1 activity increase following IPC [19,20].

**Figure 2:**
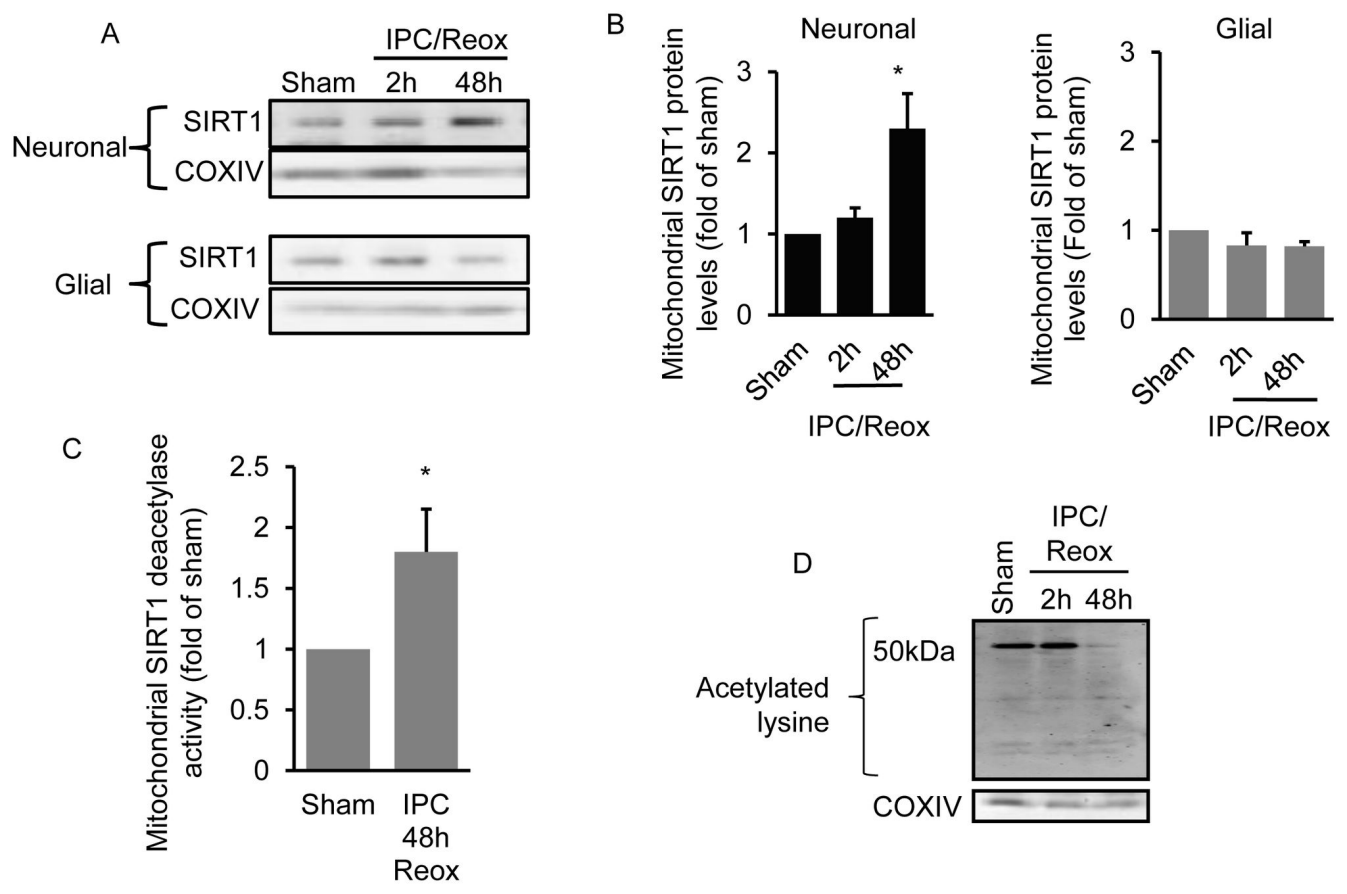
IPC increases mitochondrial SIRT1 protein levels specifically in neurons. Primary cortical glial and neuronal-only cultures were exposed to oxygen and glucose deprivation for 45 min (IPC) and the level of mitochondrial SIRT1 was determined 2 and 48 hrs later (n = 6) (A). Western blot quantitation is shown in (B). SIRT1-specific deacetylase activity was determined in mitochondrial lysates from neuronal cultures 48 hrs following exposure to sham or IPC (n = 4) (C). Neuronal mitochondria acetylation levels were determined at 2 and 48 hrs following IPC exposure (n =3) (D). * *p* < 0.05 increase from sham by one-way analysis of variance (ANOVA) and Dunnetts’ multiple comparison post hoc test.

Next, we determined whether changes in mitochondrial SIRT1 protein levels were neuronal-specific or whether it occurred in all brain mitochondria. To answer this question, we generated primary cortical neuronal-only or glial-only cultures and examined mitochondrial SIRT1 protein levels after exposure to in vitro IPC, as described in methods. Similar to our results *in vivo* in the hippocampus, mitochondrial SIRT1 protein levels were significantly (*p* < 0.05) increased at 48 hrs but not at 2 hrs following IPC exposure in neuronal-only cultures (Figure 2A and B). There was no change in mitochondrial SIRT1 protein levels in glial-only cultures at any of the time points examined. The increase in neuronal-mitochondrial SIRT1 protein levels correlated with an increase (*p* < 0.05) in mitochondrial SIRT1-specific deacetylase activity (Figure 2C) and with a reduction in acetylation of a 50 kDa mitochondrial protein (Figure 2D).

**Figure 3:**
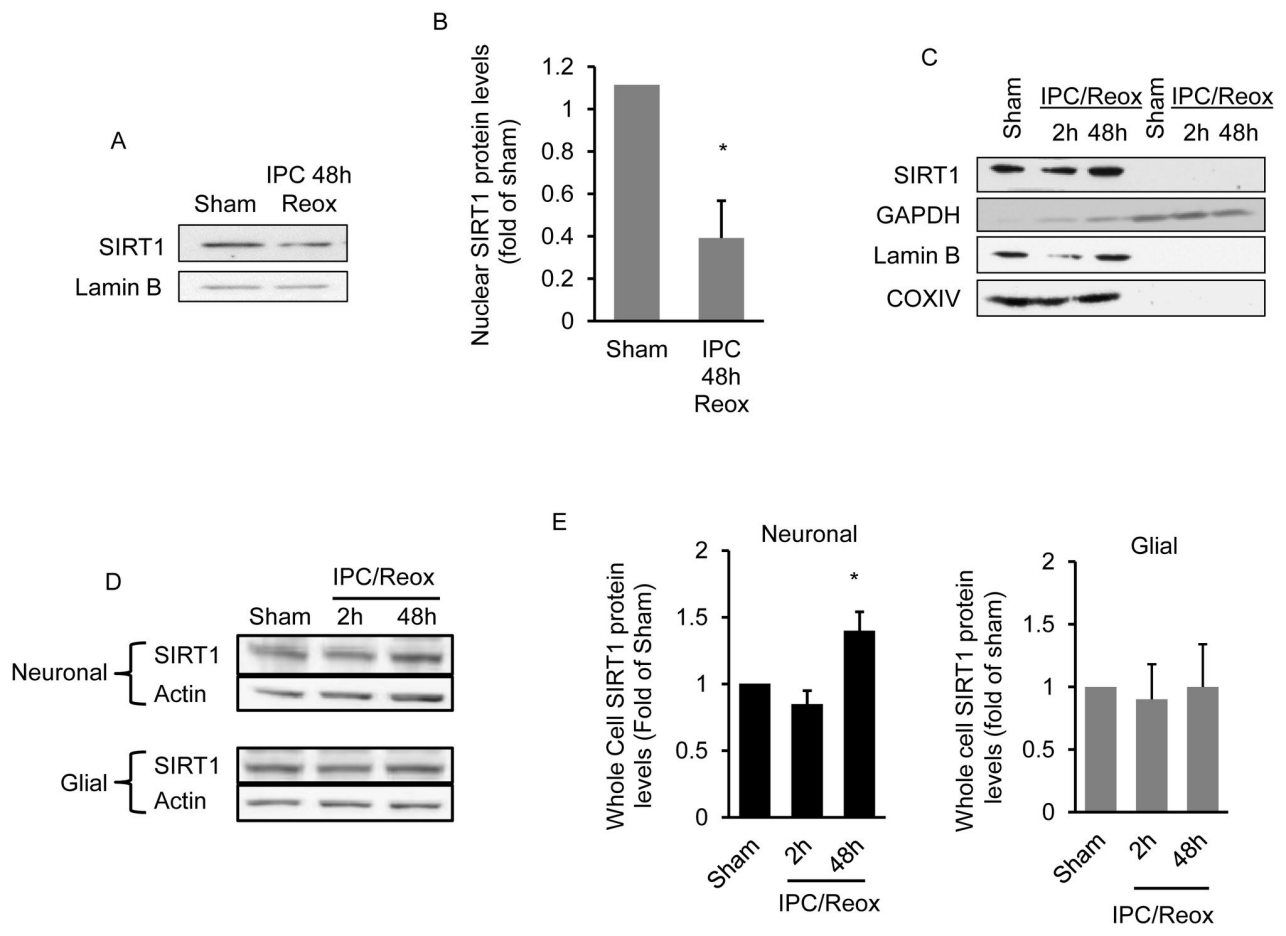
IPC increases SIRT1 expression and decreases nuclear SIRT1 protein levels. Cortical neuronal cultures were exposed to oxygen and glucose deprivation for 45 min (IPC) and nuclear SIRT1 protein levels determined 48 hrs later (n = 5) (A). Western blot quantitation of nuclear SIRT1 protein levels is shown in (B). The effect of IPC on cytoplasmic SIRT1 protein levels is shown in (C). The soluble cytoplasmic fraction was isolated from the nuclear and mitochondrial fractions as described in methods. The purity of the cytoplasmic fraction was determined by reprobing the blot for the cytoplasmic marker, GAPDH, with the nuclear marker lamin B and with the mitochondrial marker COXIV. In (D) whole cell SIRT1 protein levels in cortical glial and neuronal only cultures are shown at 2 and 48 hrs following IPC exposure (n=8). Western blot quantitation is shown in (E). Data are means ± SEM compared to sham treated animals. * *p* < 0.05 increase from sham by one-way analysis of variance (ANOVA) and Dunnetts’ multiple comparison post hoc test.

Next we determined if the increase in mitochondrial SIRT1 is the result of increased SIRT1 expression or the translocation of SIRT1 to the mitochondria from the nuclear or cytoplasmic compartments. Using subcellular fractionation we observed a significant (*p* < 0.05) reduction in nuclear SIRT1 protein levels 48 hrs following IPC exposure (Figure 3A and B). SIRT1 was not observed in the cytoplasm at any of the time points examined (Figure 3C). IPC exposure was also found to significantly (*p* < 0.05) increase total cellular SIRT1 protein levels in neuronal but not glial only-cultures (Figure 3D and E). These results indicate that SIRT1 is specifically targeted to neuronal mitochondria when SIRT1 is activated by brief ischemia/reperfusion.

### Epsilon PKC regulates mitochondrial SIRT1 protein levels

**Figure 4:**
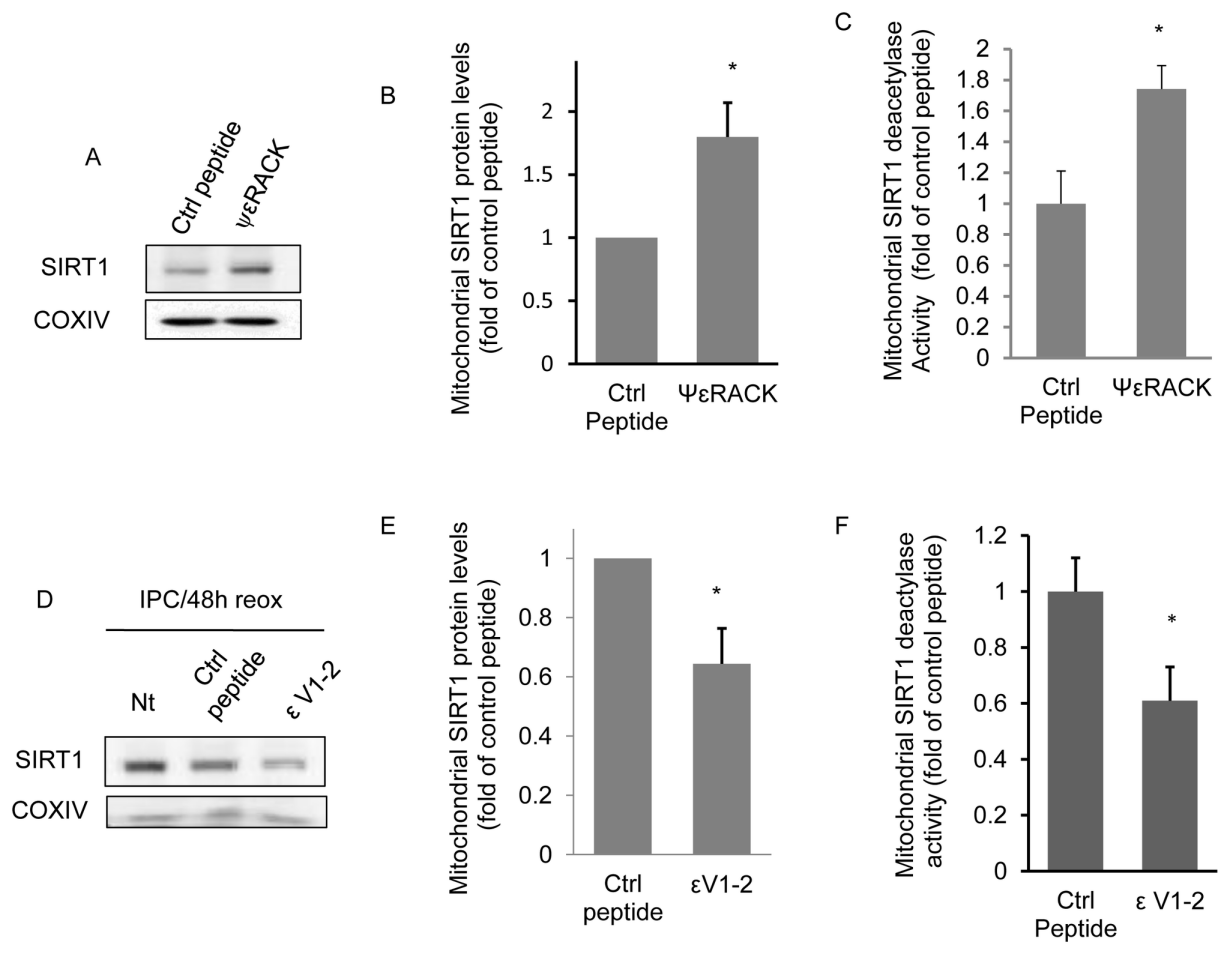
Epsilon PKC regulates mitochondrial SIRT1 protein levels. Neuronal cultures were treated with the PKCε activator ψεRACK (100 nM) or with the carrier TAT control peptide (100 nM) for 1 hr and mitochondrial SIRT1 protein levels (A and B) and deacetylase activity (C) determined 48 hrs later (n = 4). In (D) PKCε activation was inhibited with the PKCε specific inhibitor ɛV1-2 (100 nM) during and following 45 min of oxygen and glucose deprivation (IPC) and the level of mitochondrial SIRT1 determined 48 hrs later (n = 3) in non-treated (Nt), control peptide and ɛV1-2 treated samples. Western blot quantitation is shown in (E). The effect of ɛV1-2 (100 nM) on IPC induced mitochondrial SIRT1 deacetylase activity is shown in (F) (n =4). Data are means ± SEM compared to control (Ctrl) TAT peptide treated cultures. * *p* < 0.05 increase from controls by Student’s *t*-test.

Next, we were interested in identifying the mechanism by which SIRT1 is targeted to the mitochondria. Our laboratory and others have demonstrated that a novel type protein kinase C, PKCε, is both required and sufficient for IPC-induced ischemic protection [8,10,24,32]. We also demonstrated that PKCε translocates to mitochondria following IPC and promoted mitochondrial protection against cerebral ischemia [9]. In the ischemia/reperfused heart, mitochondrial PKCε protein levels are increased, and required for cardioprotection [24]. Therefore, we tested the hypothesis that PKCε mediates the increases in mitochondrial SIRT1 protein levels following IPC. To test this hypothesis, we activated PKCε in cortical neuronal cultures with ψεRACK (100 nM), a specific PKCε activator [33], and observed a significant (*p* < 0.05) increase in both mitochondrial SIRT1 protein levels and SIRT1 deacetylase activity 48 hrs later (Figure 4A-C). To confirm that PKCε is required for increasing mitochondrial SIRT1 protein levels following IPC, we exposed neuronal cultures to the PKCε inhibitor (εV1-2, 100 nM [34]) or control TAT peptide during and following IPC exposure. As demonstrated in Figure 4 (D–F), 48 hours following IPC exposure, the increase in mitochondrial SIRT1 protein levels and SIRT1 deacetylase activity was significantly (*p* < 0.05) reduced by PKCε inhibition. These results confirm our hypothesis that PKCε increases the level and activity of mitochondrial SIRT1 in neurons.

### Heat shock protein 90 is required for mitochondrial import of SIRT1

**Figure 5:**
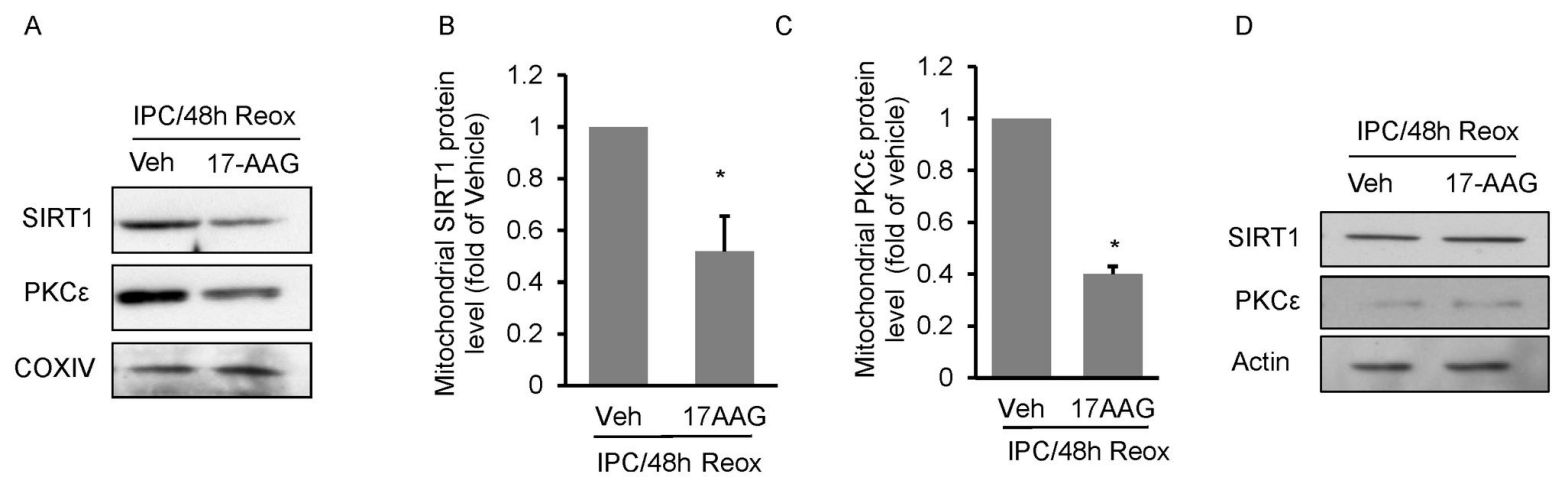
HSP90 is required for SIRT1 mitochondrial import. Mitochondrial SIRT1 and PKCε protein levels were determined, 48 hrs following IPC exposure, in neuronal cultures treated with the HSP90 inhibitor, 17-AAG (100 nM) (A). Western blot quantitation is shown in (B and C; n = 3). The effects of 17-AAG treatment on whole cell SIRT1 and PKCε are shown in (D). Data are means ± SEM compared to vehicle (Veh) treated cultures. * *p* < 0.05 increase from controls by Student’s *t*-test.

SIRT1 was shown to contain both nuclear import and export signal sequences [35], but not a mitochondrial targeting sequence. Therefore, we were interested in determining how SIRT1 was targeted into the mitochondria. In the heart, PKCε which lacks a mitochondrial targeting sequence, requires the activity of the stress chaperone protein, heat shock protein 90 (HSP90), for mitochondrial import [24]. Therefore, we hypothesized that mitochondrial import of SIRT1 requires HSP90. To test this hypothesis, we treated neuronal cultures with the HSP90 inhibitor 17-AAG (100 nM) [36], following IPC exposure. As illustrated in Figure 5 (A-C), HSP90 inhibition significantly (*p* < 0.05) reduced mitochondrial SIRT1 and PKCε protein levels 48 hrs following IPC exposure. 17-AAG treatment had no effect on total cellular SIRT1 or PKCε protein levels (Figure 5D), indicating the reduction in mitochondrial SIRT1 and PKCε was not the result of altered expression or degradation of either protein, but rather inhibition of transport of both SIRT1 and PKCε into the mitochondria.

### SIRT1 and PKCε localize within mitoplasts

**Figure 6:**
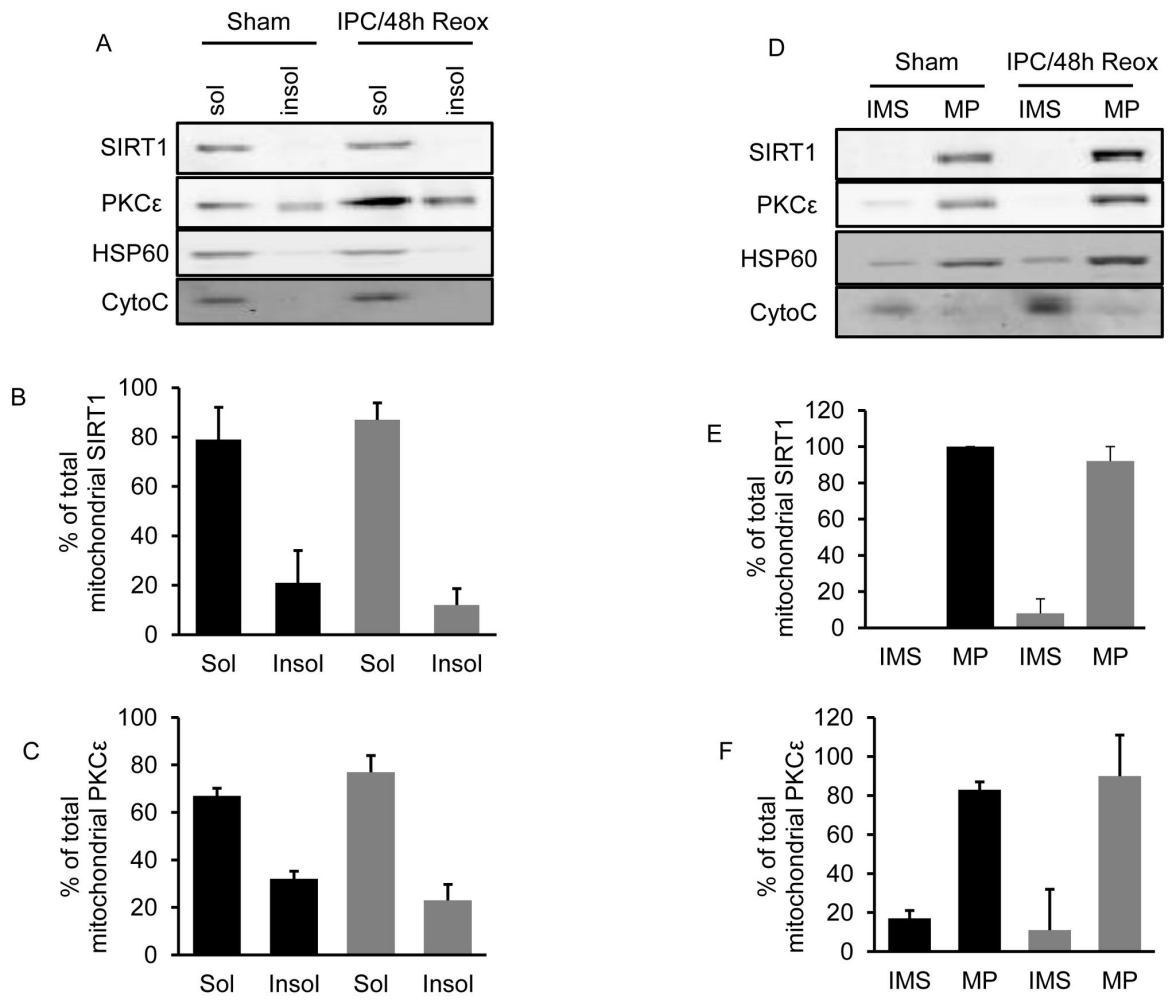
SIRT1 localizes to the mitoplast fraction. Mitochondria from sham or IPC-treated neuronal cultures were exposed to alkaline extraction and the localization of SIRT1, PKCε, cytochrome c (an intermembrane space marker), and HSP60 (a matrix marker) was determined by Western blot analysis (A). Western blot quantitation of SIRT1 and PKCε levels in the soluble (sol) and insoluble (Insol) fractions are shown in (B and C) (n = 3). In (D), SIRT1 and PKCε localizations were determined in mitoplasts (MP) and intermembrane space (IMS) fractions generated by hypoosmotic lysis of the outer mitochondrial membrane. Western blot quantitation of SIRT1 and PKCε levels in MP and IMS fractions are shown in (E and F) (n = 3). No significant difference (p > 0.05) was observed in the localization of SIRT1 or PKCε following IPC exposure when compared to sham controls. Data are means ± SEM.

To determine the submitochondrial localization of SIRT1, we exposed mitochondria to alkaline extraction which removes soluble and peripheral but not integral membrane proteins. SIRT1 from both sham and IPC treated cultures was found to primarily localize with cytochrome c and HSP60 in the supernatant following alkaline treatment (Figure 6A and B). A small portion of mitochondrial SIRT1 was also observed in the insoluble fraction. Similarly, PKCε was found primarily but not exclusively in the supernatant (Figure 6A and C). Exposure to IPC did not significantly alter the levels of SIRT1 or PKCε found in the soluble or insoluble fraction when compared to sham control. These results suggest that the majority of SIRT1 and PKCε are not strongly bound to mitochondrial membrane. Next, we used hypoosmotic lysis of the outer mitochondrial membrane to generate mitoplasts which consists of an intact mitochondrial inner membrane and matrix. After mitoplast generation the intermembrane space protein, cytochrome c, was found in the postmitoplast supernatant whereas SIRT1 was found to almost exclusively localize with the matrix protein, HSP60, in the mitoplast (Figure 6D and E). Exposing cultures to IPC did not significantly alter the submitochondrial localization of SIRT1 in the matrix or intermembrane space compared to sham treated cultures. Similarly, PKCε was found predominantly in the mitoplast fraction with a small portion of PKCε protein observed in the postmitoplast supernatant (Figure 6D and F). The submitochondrial localization of PKCε following IPC exposure was not significantly different then sham treated cultures. The finding of PKCε in the intermembrane space is consistent with data from Costa et al. [37] which proposes that mitochondrial PKCε exist in two populations, one in the intermembrane space and the other in the matrix. Both populations of PKCε coordinate in the opening of the ATP-sensitive K^+^ channels (mitoK_ATP_) and mitochondrial neuroprotection.

### Mitochondria respiration is not regulated by mitochondrial SIRT1 deacetylase activity

**Figure 7:**
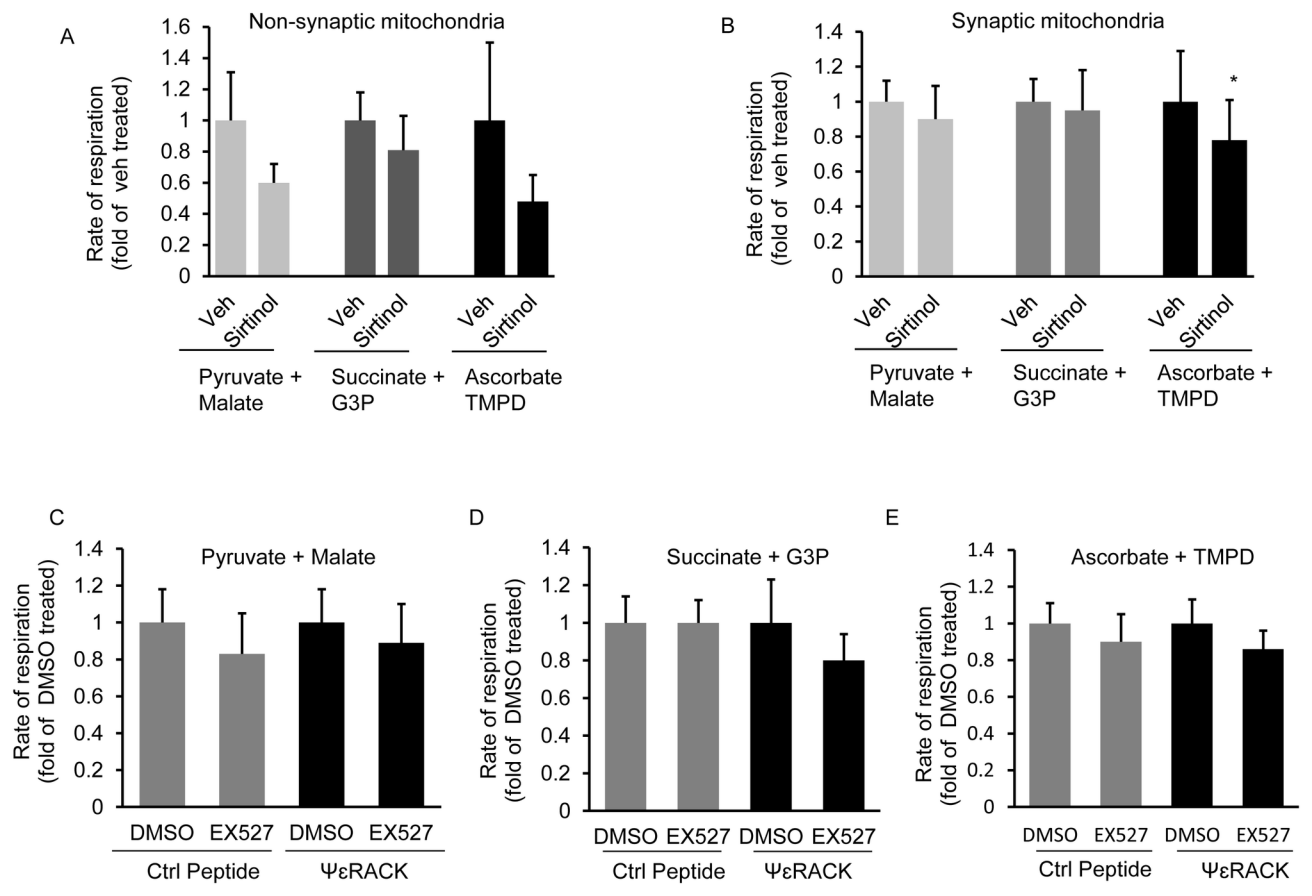
Inhibition of mitochondrial SIRT1 deacetylase activity does not alter mitochondrial respiration rate. Cortical non-synaptic (A) and synaptic (B) mitochondria were isolated from naive rats and treated with either DMSO (Veh) or the sirtuin inhibitor, sirtinol and the rate of mitochondrial substrate oxidation determined (n = 5). In C-E, rats were treated with ψεRACK, to induce PKCε preconditioning, or control peptide (Ctrl) and the non-synaptic mitochondria isolated 48 hrs later. Non-synaptic mitochondria were treated with the specific SIRT1 inhibitor, EX527, or DMSO and the rate of mitochondrial substrate oxidation determined (n = 5). Data are means ± SEM compared to veh treated groups. * *p* = 0.019 increase from vehicle controls by paired Student’s *t*-test.

Recent research has demonstrated that numerous mitochondrial proteins associated with energy metabolism are acetylated [38] [39]. In the heart, caloric restriction, a known activator of the sirtuin family, primes mitochondria for ischemic stress by deacetylating specific proteins of the electron transport chain [40]. Since our results demonstrate that SIRT1 localizes within mitoplasts, which would place it in a position to interact with proteins of the electron transport chain, we hypothesized that mitochondrial SIRT1 may regulate mitochondrial respiration following preconditioning. As a first step to test this hypothesis we isolated cortical synaptic and non-synaptic mitochondria from naive rats and treated the isolated mitochondria with the sirtuin deacetylase inhibitor, sirtinol (1 µM). We chose to treat isolated mitochondria with sirtuin inhibitors so as to avoid any indirect effects of nuclear SIRT1 inhibition on mitochondrial function. As illustrated in Figure 7A and B, although not significant, sirtinol treatment was observed to reduce the average non-synaptic mitochondrial respiration 40%, 19%, and 52% in the presence of pyruvate plus malate, succinate plus glycerol-3-phosphate and ascorbate plus TMPD, respectively, when compared to vehicle controls. The effect of sirtinol treatment on synaptic mitochondrial respiration was less pronounced as that observed in non-synaptic mitochondria; with a reduction in mitochondrial respiration of 10% and 5% in the presence of pyruvate plus malate and succinate plus glycerol-3-phosphate, respectively and with a significant (*p* = 0.019) 22% reduction in mitochondrial respiration in the presence of ascorbate plus TMPD, when compare to vehicle controls. This data indicates a trend for reduced respiration in non-synaptic mitochondria in the presence of a sirtuin inhibitor. Therefore we hypothesized that the increase in non-synaptic mitochondrial SIRT1 protein levels observed following IPC or PKCε activation may have a greater and thus significant effect in regulating non-synaptic mitochondrial respiration during conditions of ischemic tolerance. To test this hypothesis we pretreated rats with the PKCε activator, ψεRACK, which induces a preconditioning response, or control peptide [10] [8], and isolated non-synaptic mitochondria 48 hrs later. SIRT1 specific deacetylase activity was inhibited in the isolated mitochondria by treatment with EX527 (10 µM) and the rate of mitochondrial respiration determined. As demonstrated in Figure 7C-E, EX527 treatment reduced mitochondrial respiration to similar levels in both control peptide and PKCε treated animals when compared to vehicle controls. However the reduction in mitochondrial respiration in the presence of EX527 was not significantly different in any of the conditions examined.

## Discussion

In this study, we demonstrate a novel regulatory mechanism in targeting SIRT1 to the mitochondria which we believe represents an expansion of the mechanisms and targets by which SIRT1 mediates neuroprotection. We also demonstrate that changes in mitochondrial SIRT1 protein levels were dependent upon PKCε activation, suggesting a novel interaction between the PKCε and SIRT1 signaling pathways.

Mitochondrial increases in SIRT1 protein levels following IPC were only observed during the late phase or delayed window of neuroprotection. The increase in mitochondrial SIRT1 protein is also correlated with our previously reported findings of a late phase but not early increase in nuclear SIRT1 activity [19,20]. Activation of SIRT1 during IPC is known to be protective against ischemia in both the heart and brain [19,20,41-43]. In our laboratory pharmacological inhibition of SIRT1, which would presumably antagonize both nuclear and mitochondrial SIRT1 activity, blocked IPC induced ischemic tolerance [19,20].

Although a basal level of SIRT1 protein was observed in all of the mitochondrial preparations investigated, we found that IPC only increased mitochondrial SIRT1 protein levels in non-synaptic mitochondria and in mitochondria from neuronal-only cultures. These results suggest that IPC alters SIRT1 levels primarily in mitochondria localized to neuronal cell bodies. This specific targeting of SIRT1 to a subpopulation of neuronal mitochondria may be explained by our findings that HSP90 is required for mitochondrial import of SIRT1. In the adult rat brain, HSP90 is reported to primarily localize to the neuronal cell body [44]. Gass et al. [45] showed that HSP90 immunoreactivity was found in all neuronal populations of the hippocampus and neocortex, and that it was found to be primarily perikarya in location and was found to be absent from glia and from synaptic sites. These findings on HSP90 localization correlate well with our findings in mitochondrial SIRT1 protein levels following IPC.

We also demonstrate that the total cellular SIRT1 protein levels were increased following IPC exposure in neuronal but not glial only-cultures which correlates with the observed increases in neuronal but not glial mitochondrial SIRT1 protein levels. We also observed a reduction in nuclear SIRT1 protein levels 48 hrs following IPC exposure. These findings suggest that the increase in mitochondrial SIRT1 protein levels may be the result of both increased SIRT1 expression, as well as, nuclear to mitochondrial shuttling of SIRT1. Our data also demonstrate that SIRT1 is targeted to mitochondria by mechanisms mediated by PKCε. This is based upon our findings that activation of PKCε, in the absence of IPC, increases mitochondrial SIRT1 protein levels and deacetylase activity, whereas inhibition of PKCε prevents increases in mitochondrial SIRT1 protein levels and deacetylase activity following IPC exposure. The subcellular localization of SIRT1 is known to be regulated by posttranslational phosphorylation and sumoylation [35,46,47]. In the heart, desumoylation of SIRT1 induces a nuclear-to-cytoplasmic shuttling of SIRT1, whereas phosphorylation of SIRT1 by phosphoinositide 3-hydroxykinase (PI3K)-AKT in C2C12 cells induces cytoplasmic-to-nuclear translocation of SIRT1 [46]. In the above mentioned studies, the neuroprotective function of SIRT1, against ischemia/reperfusion injury and oxidative stress, was only observed when SIRT1 was located in the nucleus but not in the cytoplasm. Similarly, in endothelial cells, cytoplasmic-to-nuclear shuttling of SIRT1 is required for protection against high glucose induced apoptosis [48]. Therefore, targeting of SIRT1 to the mitochondria by PKCε may be required for IPC mediated neuroprotection. This hypothesis is supported by the fact that PKCε activation, which increases mitochondrial SIRT1 protein levels, is critical for IPC neuroprotection against ischemic exposure [7,8,10]. The mechanism by which PKCε regulates mitochondrial SIRT1 protein levels is unclear, but most likely involves either direct phosphorylation of SIRT1 by PKCε or posttranslational modification of SIRT1 by a PKCε dependent signaling cascade. Future studies are required to better delineate the interactions of SIRT1 and PKCε. The physiological role of SIRT1 in the mitochondria under both normal physiological conditions and under stress conditions remains undefined. In these studies, we found that acute inhibition of mitochondrial SIRT1 deacetylase activity had no significant effect on non-synaptic mitochondrial respiration, suggesting a possible role for SIRT1 in regulating mitochondrial gene expression. In the nucleus SIRT1 directly regulates the activity of peroxisome proliferator-activated receptor γ co-activator 1α (PGC-1α); a major transcriptional co-activator of genes involved in energy metabolism [49-51]. A similar association of SIRT1 and PGC-1α was also observed in the mitochondria by Aquilano et al. [21]. Therefore, we reason that IPC-induced changes in both nuclear and mitochondrial SIRT1 activity may allow for a coordination of mitochondrial protein expression from both the nuclear and mitochondrial genomes. Further support for this hypothesis comes from Aquilano et al. [21] studies that showed mitochondrial SIRT1 was associated with mitochondrial DNA and the transcription factor TFAM. Two important nuclear transcription factors, PGC-1α and nuclear respiratory factor-1 (NRF-1) when upregulated, lead to transcription of TFAM, which plays a key role in mitochondrial DNA replication (reviewed in [52]). Thus, we propose that IPC-induced SIRT1 translocation to the nucleus and mitochondria serves as a signaling pathway that orchestrates upregulation of electron transport subunits that may be incorporated into preexisting mitochondrial network (reviewed in [52]). This fact may also explain why our results point to SIRT1 translocation to mitochondria occurs primarily in the somatic fraction of neurons and not the synaptosomal fraction.

In summary, it has been demonstrated in both the heart and brain that SIRT1 is an important mediator of IPC-induced ischemic tolerance. Based upon the data presented here, part of SIRT1 protection may stem from the ability of IPC to regulate SIRT1 mitochondrial protein levels, which may allow for a direct regulation of mitochondrial function by SIRT1. Our discovery that SIRT1 is targeted to mitochondria by PKCε following IPC may offer new therapeutic directions in the targeting of metabolic dysfunction, which is associated with the pathophysiology of numerous diseases such as ischemia/reperfusion.

## References

[1] Stagliano NE, Pérez-Pinzón MA, Moskowitz MA, Huang PL (1999) Focal ischemic preconditioning induces rapid tolerance to middle cerebral artery occlusion in mice. J Cereb Blood Flow Metab 19: 757-761. PubMed: 10413030. 10413030 10.1097/00004647-199907000-00005

[2] Pérez-Pinzón MA, Xu GP, Dietrich WD, Rosenthal M, Sick TJ (1997) Rapid preconditioning protects rats against ischemic neuronal damage after 3 but not 7 days of reperfusion following global cerebral ischemia. J Cereb Blood Flow Metab 17: 175-182. PubMed: 9040497. 9040497 10.1097/00004647-199702000-00007

[3] Kitagawa K, Matsumoto M, Tagaya M, Hata R, Ueda H et al. (1990) 'Ischemic tolerance' phenomenon found in the brain. Brain Res 528: 21-24. doi:10.1016/0006-8993(90)90189-I. PubMed: 2245337. 2245337 10.1016/0006-8993(90)90189-i

[4] Iadecola C, Anrather J (2011) Stroke research at a crossroad: asking the brain for directions. Nat Neurosci 14: 1363-1368. doi:10.1038/nn.2953. PubMed: 22030546. 22030546 10.1038/nn.2953 PMC3633153

[5] Dirnagl U, Becker K, Meisel A (2009) Preconditioning and tolerance against cerebral ischaemia: from experimental strategies to clinical use. Lancet Neurol 8: 398-412. doi:10.1016/S1474-4422(09)70054-7. PubMed: 19296922. 19296922 10.1016/S1474-4422(09)70054-7 PMC2668955

[6] Bernaudin M, Tang Y, Reilly M, Petit E, Sharp FR (2002) Brain genomic response following hypoxia and re-oxygenation in the neonatal rat. Identification of genes that might contribute to hypoxia-induced ischemic tolerance. J Biol Chem 277: 39728-39738. doi:10.1074/jbc.M204619200. PubMed: 12145288. 12145288 10.1074/jbc.M204619200

[7] Jia J, Wang X, Li H, Han S, Zu P et al. (2007) Activations of nPKCepsilon and ERK1/2 were involved in oxygen-glucose deprivation-induced neuroprotection via NMDA receptors in hippocampal slices of mice. J Neurosurg Anesthesiol 19: 18-24. doi:10.1097/01.ana.0000211020.88431.e2. PubMed: 17198096. 17198096 10.1097/01.ana.0000211020.88431.e2

[8] Raval AP, Dave KR, Mochly-Rosen D, Sick TJ, Pérez-Pinzón MA (2003) Epsilon PKC is required for the induction of tolerance by ischemic and NMDA-mediated preconditioning in the organotypic hippocampal slice. J Neurosci 23: 384-391. PubMed: 12533598. 12533598 10.1523/JNEUROSCI.23-02-00384.2003 PMC6741876

[9] Raval AP, Dave KR, DeFazio RA, Perez-Pinzon MA (2007) epsilonPKC phosphorylates the mitochondrial K(+) (ATP) channel during induction of ischemic preconditioning in the rat hippocampus. Brain Res 1184: 345-353. doi:10.1016/j.brainres.2007.09.073. PubMed: 17988655. 17988655 10.1016/j.brainres.2007.09.073 PMC2577914

[10] Kim E, Raval AP, Defazio RA, Perez-Pinzon MA (2007) Ischemic preconditioning via epsilon protein kinase C activation requires cyclooxygenase-2 activation in vitro. Neuroscience 145: 931-941. doi:10.1016/j.neuroscience.2006.12.063. PubMed: 17307294. 17307294 10.1016/j.neuroscience.2006.12.063 PMC2153455

[11] Chen D, Steele AD, Lindquist S, Guarente L (2005) Increase in activity during calorie restriction requires Sirt1. Science 310: 1641. doi:10.1126/science.1118357. PubMed: 16339438. 16339438 10.1126/science.1118357

[12] Haigis MC, Guarente LP (2006) Mammalian sirtuins--emerging roles in physiology, aging, and calorie restriction. Genes Dev 20: 2913-2921. doi:10.1101/gad.1467506. PubMed: 17079682. 17079682 10.1101/gad.1467506

[13] Nogueiras R, Habegger KM, Chaudhary N, Finan B, Banks AS et al. (2012) Sirtuin 1 and sirtuin 3: physiological modulators of metabolism. Physiol Rev 92: 1479-1514. doi:10.1152/physrev.00022.2011. PubMed: 22811431. 22811431 10.1152/physrev.00022.2011 PMC3746174

[14] de Oliveira RM, Pais TF, Outeiro TF (2010) Sirtuins: common targets in aging and in neurodegeneration. Curr Drug Targets 11: 1270-1280. doi:10.2174/1389450111007011270. PubMed: 20840069. 20840069 10.2174/1389450111007011270

[15] Jeong H, Cohen DE, Cui L, Supinski A, Savas JN et al. (2012) Sirt1 mediates neuroprotection from mutant huntingtin by activation of the TORC1 and CREB transcriptional pathway. Nat Med 18: 159-165. PubMed: 22179316. 10.1038/nm.2559 PMC3509213 22179316

[16] Jiang M, Wang J, Fu J, Du L, Jeong H et al. (2012) Neuroprotective role of Sirt1 in mammalian models of Huntington’s disease through activation of multiple Sirt1 targets. Nat Med 18: 153-158. PubMed: 22179319. 10.1038/nm.2558 PMC4551453 22179319

[17] Clark D, Tuor UI, Thompson R, Institoris A, Kulynych A et al. (2012) Protection against recurrent stroke with resveratrol: endothelial protection. PLOS ONE 7: e47792. doi:10.1371/journal.pone.0047792. PubMed: 23082218. 23082218 10.1371/journal.pone.0047792 PMC3474795

[18] Zhang T, Kraus WL (2010) SIRT1-dependent regulation of chromatin and transcription: linking NAD(+) metabolism and signaling to the control of cellular functions. Biochim Biophys Acta 1804: 1666-1675. doi:10.1016/j.bbapap.2009.10.022. PubMed: 19879981. 19879981 10.1016/j.bbapap.2009.10.022 PMC2886162

[19] Della-Morte D, Dave KR, DeFazio RA, Bao YC, Raval AP et al. (2009) Resveratrol pretreatment protects rat brain from cerebral ischemic damage via a sirtuin 1-uncoupling protein 2 pathway. Neuroscience 159: 993-1002. doi:10.1016/j.neuroscience.2009.01.017. PubMed: 19356683. 19356683 10.1016/j.neuroscience.2009.01.017 PMC2668125

[20] Raval AP, Dave KR, Pérez-Pinzón MA (2006) Resveratrol mimics ischemic preconditioning in the brain. J Cereb Blood Flow Metab 26: 1141-1147. PubMed: 16395277. 16395277 10.1038/sj.jcbfm.9600262

[21] Aquilano K, Vigilanza P, Baldelli S, Pagliei B, Rotilio G et al. (2010) Peroxisome proliferator-activated receptor gamma co-activator 1alpha (PGC-1alpha) and sirtuin 1 (SIRT1) reside in mitochondria: possible direct function in mitochondrial biogenesis. J Biol Chem 285: 21590-21599. doi:10.1074/jbc.M109.070169. PubMed: 20448046. 20448046 10.1074/jbc.M109.070169 PMC2898414

[22] Dave KR, Lange-Asschenfeldt C, Raval AP, Prado R, Busto R et al. (2005) Ischemic preconditioning ameliorates excitotoxicity by shifting glutamate/gamma-aminobutyric acid release and biosynthesis. J Neurosci Res 82: 665-673. doi:10.1002/jnr.20674. PubMed: 16247804. 16247804 10.1002/jnr.20674

[23] Dave KR, DeFazio RA, Raval AP, Torraco A, Saul I et al. (2008) Ischemic preconditioning targets the respiration of synaptic mitochondria via protein kinase C epsilon. J Neurosci 28: 4172-4182. doi:10.1523/JNEUROSCI.5471-07.2008. PubMed: 18417696. 18417696 10.1523/JNEUROSCI.5471-07.2008 PMC2678917

[24] Budas GR, Churchill EN, Disatnik MH, Sun L, Mochly-Rosen D (2010) Mitochondrial import of PKCepsilon is mediated by HSP90: a role in cardioprotection from ischaemia and reperfusion injury. Cardiovasc Res 88: 83-92. doi:10.1093/cvr/cvq154. PubMed: 20558438. 20558438 10.1093/cvr/cvq154 PMC2936125

[25] Vijayvergiya C, Beal MF, Buck J, Manfredi G (2005) Mutant superoxide dismutase 1 forms aggregates in the brain mitochondrial matrix of amyotrophic lateral sclerosis mice. J Neurosci 25: 2463-2470. doi:10.1523/JNEUROSCI.4385-04.2005. PubMed: 15758154. 15758154 10.1523/JNEUROSCI.4385-04.2005 PMC6725162

[26] Horn D, Al-Ali H, Barrientos A (2008) Cmc1p is a conserved mitochondrial twin CX9C protein involved in cytochrome c oxidase biogenesis. Mol Cell Biol 28: 4354-4364. doi:10.1128/MCB.01920-07. PubMed: 18443040. 18443040 10.1128/MCB.01920-07 PMC2447134

[27] Kim EJ, Raval AP, Perez-Pinzon MA (2008) Preconditioning mediated by sublethal oxygen-glucose deprivation-induced cyclooxygenase-2 expression via the signal transducers and activators of transcription 3 phosphorylation. J Cereb Blood Flow Metab 28: 1329-1340. doi:10.1038/jcbfm.2008.26. PubMed: 18398416. 18398416 10.1038/jcbfm.2008.26 PMC2645802

[28] Caito S, Rajendrasozhan S, Cook S, Chung S, Yao H et al. (2010) SIRT1 is a redox-sensitive deacetylase that is post-translationally modified by oxidants and carbonyl stress. FASEB J 24: 3145-3159. doi:10.1096/fj.09-151308. PubMed: 20385619. 20385619 10.1096/fj.09-151308 PMC2923349

[29] Bright R, Sun GH, Yenari MA, Steinberg GK, Mochly-Rosen D (2008) epsilonPKC confers acute tolerance to cerebral ischemic reperfusion injury. Neurosci Lett 441: 120-124. doi:10.1016/j.neulet.2008.05.080. PubMed: 18586397. 18586397 10.1016/j.neulet.2008.05.080 PMC2597630

[30] Della-Morte D, Raval AP, Dave KR, Lin HW, Perez-Pinzon MA (2011) Post-ischemic activation of protein kinase C epsilon protects the hippocampus from cerebral ischemic injury via alterations in cerebral blood flow. Neurosci Lett 487: 158-162. doi:10.1016/j.neulet.2010.10.013. PubMed: 20951185. 20951185 10.1016/j.neulet.2010.10.013 PMC3004991

[31] Napper AD, Hixon J, McDonagh T, Keavey K, Pons JF et al. (2005) Discovery of indoles as potent and selective inhibitors of the deacetylase SIRT1. J Med Chem 48: 8045-8054. doi:10.1021/jm050522v. PubMed: 16335928. 16335928 10.1021/jm050522v

[32] Liu GS, Cohen MV, Mochly-Rosen D, Downey JM (1999) Protein kinase C-epsilon is responsible for the protection of preconditioning in rabbit cardiomyocytes. J Mol Cell Cardiol 31: 1937-1948. doi:10.1006/jmcc.1999.1026. PubMed: 10525430. 10525430 10.1006/jmcc.1999.1026

[33] Dorn GW 2nd, Souroujon MC, Liron T, Chen CH, Gray MO et al. (1999) Sustained in vivo cardiac protection by a rationally designed peptide that causes epsilon protein kinase C translocation. Proc Natl Acad Sci U S A 96: 12798-12803. doi:10.1073/pnas.96.22.12798. PubMed: 10536002. 10536002 10.1073/pnas.96.22.12798 PMC23103

[34] Johnson JA, Gray MO, Chen CH, Mochly-Rosen D (1996) A protein kinase C translocation inhibitor as an isozyme-selective antagonist of cardiac function. J Biol Chem 271: 24962-24966. doi:10.1074/jbc.271.40.24962. PubMed: 8798776. 8798776 10.1074/jbc.271.40.24962

[35] Tanno M, Sakamoto J, Miura T, Shimamoto K, Horio Y (2007) Nucleocytoplasmic shuttling of the NAD^+^-dependent histone deacetylase SIRT1. J Biol Chem 282: 6823-6832. PubMed: 17197703. 17197703 10.1074/jbc.M609554200

[36] Schulte TW, Neckers LM (1998) The benzoquinone ansamycin 17-allylamino-17-demethoxygeldanamycin binds to HSP90 and shares important biologic activities with geldanamycin. Cancer Chemother Pharmacol 42: 273-279. doi:10.1007/s002800050817. PubMed: 9744771. 9744771 10.1007/s002800050817

[37] Costa AD, Garlid KD (2008) Intramitochondrial signaling: interactions among mitoKATP, PKCepsilon, ROS, and MPT. Am J Physiol Heart Circ Physiol 295: H874-H882. doi:10.1152/ajpheart.01189.2007. PubMed: 18586884. 18586884 10.1152/ajpheart.01189.2007 PMC2519212

[38] Kim SC, Sprung R, Chen Y, Xu Y, Ball H et al. (2006) Substrate and functional diversity of lysine acetylation revealed by a proteomics survey. Mol Cell 23: 607-618. doi:10.1016/j.molcel.2006.06.026. PubMed: 16916647. 16916647 10.1016/j.molcel.2006.06.026

[39] Choudhary C, Kumar C, Gnad F, Nielsen ML, Rehman M et al. (2009) Lysine acetylation targets protein complexes and co-regulates major cellular functions. Science 325: 834-840. doi:10.1126/science.1175371. PubMed: 19608861. 19608861 10.1126/science.1175371

[40] Shinmura K, Tamaki K, Sano M, Nakashima-Kamimura N, Wolf AM et al. (2011) Caloric restriction primes mitochondria for ischemic stress by deacetylating specific mitochondrial proteins of the electron transport chain. Circ Res 109: 396-406. doi:10.1161/CIRCRESAHA.111.243097. PubMed: 21700931. 21700931 10.1161/CIRCRESAHA.111.243097

[41] Nadtochiy SM, Yao H, McBurney MW, Gu W, Guarente L et al. (2011) SIRT1-mediated acute cardioprotection. Am J Physiol Heart Circ Physiol 301: H1506-H1512. doi:10.1152/ajpheart.00587.2011. PubMed: 21856913. 21856913 10.1152/ajpheart.00587.2011 PMC3197366

[42] Nadtochiy SM, Redman E, Rahman I, Brookes PS (2011) Lysine deacetylation in ischaemic preconditioning: the role of SIRT1. Cardiovasc Res 89: 643-649. doi:10.1093/cvr/cvq287. PubMed: 20823277. 20823277 10.1093/cvr/cvq287 PMC3028968

[43] Shinmura K, Tamaki K, Bolli R (2008) Impact of 6-mo caloric restriction on myocardial ischemic tolerance: possible involvement of nitric oxide-dependent increase in nuclear Sirt1. Am J Physiol Heart Circ Physiol 295: H2348-H2355. doi:10.1152/ajpheart.00602.2008. PubMed: 18931029. 18931029 10.1152/ajpheart.00602.2008 PMC2614541

[44] Itoh H, Tashima Y, Eishi Y, Okeda R (1993) Localization of HSP90 in rat brain. Int J Biochem 25: 93-99. doi:10.1016/0020-711X(93)90494-Y. PubMed: 8432386. 8432386 10.1016/0020-711x(93)90494-y

[45] Gass P, Schröder H, Prior P, Kiessling M (1994) Constitutive expression of heat shock protein 90 (HSP90) in neurons of the rat brain. Neurosci Lett 182: 188-192. doi:10.1016/0304-3940(94)90794-3. PubMed: 7715807. 7715807 10.1016/0304-3940(94)90794-3

[46] Tong C, Morrison A, Mattison S, Qian S, Bryniarski M et al. (2012) Impaired SIRT1 nucleocytoplasmic shuttling in the senescent heart during ischemic stress. FASEB J Epub ahead of print. PubMed: 23024374. 10.1096/fj.12-216473 PMC3804750 23024374

[47] Nasrin N, Kaushik VK, Fortier E, Wall D, Pearson KJ et al. (2009) JNK1 phosphorylates SIRT1 and promotes its enzymatic activity. PLOS ONE 4: e8414. doi:10.1371/journal.pone.0008414. PubMed: 20027304. 20027304 10.1371/journal.pone.0008414 PMC2793009

[48] Hou J, Chong ZZ, Shang YC, Maiese K (2010) Early apoptotic vascular signaling is determined by Sirt1 through nuclear shuttling, forkhead trafficking, bad, and mitochondrial caspase activation. Curr Neurovasc Res 7: 95-112. doi:10.2174/156720210791184899. PubMed: 20370652. 20370652 10.2174/156720210791184899 PMC2876719

[49] Rodgers JT, Lerin C, Gerhart-Hines Z, Puigserver P (2008) Metabolic adaptations through the PGC-1 alpha and SIRT1 pathways. FEBS Lett 582: 46-53. doi:10.1016/j.febslet.2007.11.034. PubMed: 18036349. 18036349 10.1016/j.febslet.2007.11.034 PMC2275806

[50] Nemoto S, Fergusson MM, Finkel T (2005) SIRT1 functionally interacts with the metabolic regulator and transcriptional coactivator PGC-1{alpha}. J Biol Chem 280: 16456-16460. doi:10.1074/jbc.M501485200. PubMed: 15716268. 15716268 10.1074/jbc.M501485200

[51] Knutti D, Kralli A (2001) PGC-1, a versatile coactivator. Trends Endocrinol Metab 12: 360-365. doi:10.1016/S1043-2760(01)00457-X. PubMed: 11551810. 11551810 10.1016/s1043-2760(01)00457-x

[52] Perez-Pinzon MA, Stetler RA, Fiskum G (2012) Novel mitochondrial targets for neuroprotection. J Cereb Blood Flow Metab 32: 1362-1376. doi:10.1038/jcbfm.2012.32. PubMed: 22453628. 22453628 10.1038/jcbfm.2012.32 PMC3390821

